# Depression and/or Anxiety and Health-Related Quality of Life Among Adults with Anemia: A National Analysis in the United States

**DOI:** 10.3390/jcm15207884

**Published:** 2026-10-13

**Authors:** Monira Alwhaibi

**Affiliations:** Department of Clinical Pharmacy, College of Pharmacy, King Saud University, Riyadh 11149, Saudi Arabia; malwhaibi@ksu.edu.sa

**Keywords:** anemia, depression, anxiety, quality of life, mental health, comorbidity, adults, United States, patient-reported outcome measures

## Abstract

**Background:** Anemia frequently coexists with depression and anxiety, which may further compromise health-related quality of life (HRQoL). However, limited evidence exists on the independent association of these mental health comorbidities with physical and mental HRQoL among adults with anemia. The study objective is to examine the association between coexisting depression and/or anxiety and HRQoL among adults with anemia in the United States. **Methods:** A cross-sectional study was conducted among 835 adults with anemia. Participants were categorized into two groups: anemia only and anemia with depression and/or anxiety. HRQoL was assessed using the Physical Component Summary (PCS) and Mental Component Summary (MCS) scores of the Veterans RAND 12-Item Health Survey (VR-12©). Weighted descriptive statistics were used to characterize the study population, and differences between groups were evaluated using chi-square tests and t-tests. Multivariable linear regression analyses were performed to examine the association between the anemia groups and HRQoL after adjusting for sociodemographic characteristics, health insurance, general health status, physical activity, and chronic comorbidities. **Results:** Among the adults with anemia, 69.3% had anemia only and 30.7% had anemia with depression and/or anxiety. Participants with depression and/or anxiety had significantly lower mean PCS (39.61 vs. 42.78, *p* < 0.0001) and MCS (44.24 vs. 52.49, *p* < 0.0001) scores than those with anemia alone. In adjusted analyses, coexisting depression and/or anxiety was independently associated with significantly lower MCS scores (β = −7.09, SE = 0.91, *p* < 0.001) but was not associated with PCS (β = 1.04, SE = 1.00, *p* > 0.05). Poor self-rated health was strongly associated with lower PCS and MCS scores, while younger age, employment, and regular physical activity were associated with better physical HRQoL. **Conclusions:** Among adults with anemia, coexisting depression and/or anxiety was independently associated with substantially poorer mental health-related quality of life, but not with physical health-related quality of life after adjustment for sociodemographic characteristics, healthcare access, health status, physical activity, and chronic comorbidities. These findings highlight the importance of recognizing and addressing coexisting mental health conditions in adults with anemia as part of comprehensive, patient-centered care.

## 1. Introduction

Anemia is one of the most common hematological disorders worldwide and remains a major public health concern affecting individuals across all age groups. It is characterized by a reduced hemoglobin concentration or red blood cell mass, resulting in impaired oxygen delivery to tissues and organs [1]. Worldwide, anemia impacts about 24.8% of the total population [2]. In the United States, anemia affects millions of adults, particularly older individuals, women, and those with chronic medical conditions [3,4]. Beyond its hematologic manifestations, anemia is associated with fatigue, weakness, reduced physical functioning, disability, productivity loss, impaired cognitive performance, increased healthcare utilization, and higher mortality, imposing a substantial clinical and economic burden on patients and healthcare systems [5,6,7,8].

The consequences of anemia extend beyond physical symptoms. Individuals with anemia frequently experience psychological distress, including depression and anxiety, which may arise from persistent fatigue, functional limitations, chronic illness, and reduced social participation [5,9,10,11,12,13]. This relationship is likely bidirectional, as depression and anxiety may both contribute to and result from anemia [14,15]. Conversely, depression and anxiety may exacerbate the burden of anemia by impairing treatment adherence, reducing self-care behaviors, increasing symptom perception, and adversely affecting health outcomes (e.g., symptom burden, functional status, and health-related quality of life) [16]. The coexistence of anemia with common mental health disorders therefore represents an important but often under-recognized clinical challenge that may adversely affect patients’ well-being.

These interrelated physical and psychological burdens may ultimately have important consequences for patients’ overall well-being, making quality of life a particularly relevant patient-centered outcome. Health-related quality of life (HRQoL) captures the impact of disease on physical, psychological, and social functioning [17]. It has become an increasingly important endpoint in clinical research because it reflects the patient’s perception of health beyond traditional clinical and laboratory measures. Previous studies have consistently shown that anemia is associated with poorer HRQoL across diverse national populations from Netherlands and Italy [7,18,19,20], while depression and anxiety independently contribute to substantial reductions in both physical and mental functioning [21]. Among adults with anemia in the United States, approximately 20.0–27.0% had depression and 6.0 to 7.0% had anxiety disorder [22]. Globally, studies have reported that depression affects approximately 23% of adults with anemia. Yet, these mental health conditions remain under-recognized and undertreated among individuals with anemia.

Although depression/anxiety has each been associated with poorer HRQoL in anemic patients, relatively little is known about their combined impact among adults with anemia in the general United States population. Previous studies have primarily examined the association between anemia and HRQoL rather than evaluating the additional impact of coexisting depression and/or anxiety on HRQoL among adults with anemia [18]. Moreover, much of the available evidence has focused on the elderly, above 65 years or older [19,20]. Consequently, the extent to which coexisting depression and/or anxiety are independently associated with physical and mental HRQoL among adults with anemia remains insufficiently understood. Addressing this gap may provide clinically relevant evidence to identify adults with anemia who are at greater risk of impaired HRQoL and support a more comprehensive approach to anemia care. In particular, understanding the contribution of depression and anxiety may inform psychological screening, integrated physical and mental health management, and patient-centered interventions aimed at improving quality of life and overall well-being. Therefore, the objective of this study was to examine the association between coexisting depression and/or anxiety and health-related quality of life among adults with anemia in the United States using nationally representative data from the Medical Expenditure Panel Survey (MEPS). We hypothesized that adults with anemia and coexisting depression and/or anxiety would report significantly poorer physical and mental HRQoL than those with anemia alone. To our knowledge, this is among the first nationally representative United States studies to quantify the independent association of coexisting depression and/or anxiety with both physical and mental HRQoL among adults with anemia while comprehensively adjusting for sociodemographic characteristics, healthcare access, physical activity, and multiple chronic conditions.

## 2. Methods

### 2.1. Study Design and Data

This retrospective cross-sectional study utilized data from the 2017–2022 panels of the Medical Expenditure Panel Survey (MEPS), a nationally representative survey of the United States civilian noninstitutionalized population conducted by the Agency for Healthcare Research and Quality (AHRQ). MEPS employ a stratified, multistage probability sampling design and follows participants over two consecutive years through multiple rounds of computer-assisted personal interviews [23]. The survey collects detailed information on demographic and socioeconomic characteristics, chronic medical conditions, healthcare utilization and expenditure, health insurance coverage, income, and employment. Participants self-report their medical conditions during interviews, and these conditions are subsequently coded into standardized *International Classification of Diseases, Tenth Revision, Clinical Modification* (ICD-10-CM) codes.

### 2.2. Study Population

Participants were included if they were adults, defined as individuals aged 18 years or older; had a documented diagnosis of anemia; were alive during the study period; and had complete health-related quality of life (HRQoL) data. Participants were excluded from the analytic sample if they were younger than 18 years; did not have a documented diagnosis of anemia; were not alive during the study period; or had missing or incomplete HRQoL information. Anemia was identified in the Medical Expenditure Panel Survey (MEPS) using the ICD-10-CM diagnosis codes D50 (Iron deficiency anemia) and D64 (Other anemia), which are the anemia diagnoses available in the MEPS public-use files.

### 2.3. Measures

#### 2.3.1. Outcome: Health-Related Quality of Life (HRQoL)

Health-related quality of life (HRQoL) was assessed using the Veterans RAND 12-Item Health Survey (VR-12^®^), a validated and widely used patient-reported outcome measure administered as part of the Medical Expenditure Panel Survey (MEPS) [24,25]. The VR-12^®^ was developed and validated for use among United States veterans and subsequently evaluated for use in the broader adult population. The VR-12 instrument contains 12 items, which represent eight health domains, including general health, physical functioning, role limitations due to physical health, bodily pain, vitality, role limitations due to emotional problems, mental health, and social functioning. Responses to these domains are summarized into two standardized composite measures: the Physical Component Summary (PCS), which reflects physical health status based on general health, physical functioning, role limitations due to physical health, and bodily pain; and the Mental Component Summary (MCS), which assesses mental health through vitality, role limitations due to emotional problems, mental health, and social functioning. Higher PCS and MCS scores indicate better health-related quality of life [26]. Both PCS and MCS showed high internal consistency (α > 0.80). Test–retest reliability was high for PCS (ICC = 0.78) and moderate for MCS (ICC = 0.60). PCS showed strong convergent validity, while MCS demonstrated moderate convergent validity with EQ-5D and mental health measures [26].

#### 2.3.2. Independent Variables

Depression and anxiety were identified based on the corresponding ICD-10-CM diagnostic codes recorded in the MEPS. Participants with anemia were categorized into two mutually exclusive study groups according to the presence or absence of a documented diagnosis of depression and/or anxiety. The depression and/or anxiety group included participants with a documented diagnosis of depression, anxiety, or both (anemia with coexisting depression and/or anxiety), whereas the anemia-only group included participants with anemia who had no documented diagnosis of either depression or anxiety (anemia only). Additional covariates were selected based on established evidence of their association with HRQoL among adults with anemia. These included sociodemographic characteristics (age, sex, race/ethnicity, education level, marital status, employment status, poverty status, and region of residence). Health insurance and prescription drug insurance coverage were included as indicators of healthcare access, which may influence HRQoL. Self-rated general health and physical activity were also considered because of their known associations with physical and mental well-being. Furthermore, chronic comorbidities, including heart disease, hypertension, diabetes, hyperlipidemia, asthma, chronic obstructive pulmonary disease (COPD), arthritis, gastroesophageal reflux disease (GERD), and cancer, were included in the analyses and were identified using the ICD-10-CM diagnostic codes recorded in the MEPS.

### 2.4. Statistical Analyses

Descriptive statistics were used to summarize the baseline characteristics of adults with anemia, including means, standard deviations, frequencies, and weighted percentages. PROC SURVEYFREQ was used to estimate weighted frequencies and percentages for categorical variables; PROC SURVEYMEANS was used to estimate weighted means and standard deviations for continuous variables. Chi-square tests were used to assess differences between the two anemia groups (anemia only vs. anemia with depression and/or anxiety) for categorical participant characteristics, including sociodemographic variables, health insurance, self-rated general health, physical activity, and chronic comorbidities. Differences in mean health-related quality of life (HRQoL) scores, including the Physical Component Summary (PCS) and Mental Component Summary (MCS), were evaluated using independent sample t-tests. Prior to conducting the multiple linear regression analyses, model assumptions were assessed. Linearity was evaluated by examining the relationship between the predictors and the outcome, while independence of observations was considered based on the study design. Multicollinearity was assessed using variance inflation factors (VIFs) and tolerance values. Homoscedasticity was evaluated by examining the residuals versus fitted values, and the distribution of residuals was assessed using diagnostic plots. No substantial violations of the regression assumptions were identified. Multivariable linear regression models were then fitted to examine the association between anemia group and HRQoL. Two separate multivariable linear regression models were fitted: with PCS score as the dependent variable in the first model and with MCS score as the dependent variable in the second model. The primary independent variable was anemia group, comparing participants with anemia and coexisting depression and/or anxiety with those with anemia alone. Covariates included sociodemographic characteristics, healthcare access variables, general health status, physical activity, and chronic comorbidities. PROC SURVEYREG was used to perform multivariable linear regression analyses. The general model can be expressed as HRQoL_i_ = β_0_ + β_1_(Anemia group)_i_ + β_2_(Sociodemographic characteristics)_i_ + β_3_(Healthcare access)_i_ + β_4_(Health status)_i_ + β_5_(Physical activity)_i_ + β_6_(Comorbidities)_i_ + ε_i_; where HRQoL represents either PCS or MCS. Statistical significance was assessed using a two-sided α = 0.05 threshold for the chi-square tests, independent-samples t-tests, and regression analyses. All analyses accounted for the complex sampling design of the Medical Expenditure Panel Survey (MEPS) by incorporating person-level sampling weights, strata, and primary sampling units to generate nationally representative estimates. Statistical analyses were performed using SAS version 9.4 (SAS Institute Inc., Cary, NC, USA; released in 2023).

## 3. Results

### 3.1. Characteristics of the Study Sample

A total of 835 adults with anemia were included in the analysis (Table 1). Of these, 567 participants (69.3%) had anemia alone, while 268 participants (30.7%) had anemia with coexisting depression and/or anxiety.

There were no statistically significant differences between the anemia groups according to age (*p* = 0.250), sex (*p* = 0.164), marital status (*p* = 0.148), education level (*p* = 0.248), region of residence (*p* = 0.194), employment status (*p* = 0.075), poverty status (*p* = 0.594), physical activity (*p* = 0.191), heart disease (*p* = 0.089), hypertension (*p* = 0.087), or diabetes (*p* = 0.550).

Significant differences were observed across racial/ethnic groups (*p* < 0.0001). White participants had the highest proportion of anemia with depression and/or anxiety (37.0%), compared with Latino (26.2%), African American (16.8%), and participants of other races/ethnicities (18.2%). Health insurance status differed significantly between the groups (*p* = 0.008). Participants with public insurance had a higher prevalence of anemia with depression and/or anxiety (37.6%) than those with private insurance (26.6%) or who were uninsured (26.8%). Similarly, individuals without prescription drug insurance were more likely to have anemia with depression and/or anxiety than those with prescription coverage (35.1% vs. 25.9%, *p* = 0.007).

General health status was strongly associated with anemia group (*p* < 0.0001). The proportion of participants with anemia and depression and/or anxiety increased progressively with worsening self-rated health, from 22.6% among those reporting excellent/very good health to 29.2% among those reporting good health and 40.2% among those reporting fair/poor health. Several chronic conditions were significantly more common among participants with anemia and depression and/or anxiety than among those with anemia alone. These included hyperlipidemia (36.2% vs. 27.8%, *p* = 0.038), asthma (45.9% vs. 28.4%, *p* = 0.001), COPD (46.1% vs. 29.5%, *p* = 0.025), arthritis (46.0% vs. 27.1%, *p* < 0.0001), GERD (39.7% vs. 28.2%, *p* = 0.006), and cancer (41.7% vs. 29.3%, *p* = 0.030). In contrast, no significant differences were observed for heart disease, hypertension, or diabetes.

### 3.2. Health-Related Quality of Life Scores by Anemia Groups

Table 2 presents the weighted mean health-related quality of life (HRQoL) scores among adults with anemia according to anemia group. Overall, the study sample had a mean Physical Component Summary (PCS) score of 40.43 (SD = 13.14) and a mean Mental Component Summary (MCS) score of 48.78 (SD = 10.90).

Participants with anemia only had significantly higher HRQoL scores than those with anemia and depression and/or anxiety. The mean PCS score was 42.78 (SE = 0.71) among participants with anemia only compared with 39.61 (SE = 0.98) among those with anemia and depression and/or anxiety (*p* < 0.0001). Similarly, the mean MCS score was substantially higher in the anemia-only group (52.49, SE = 0.36) than in the anemia with depression and/or anxiety group (44.24, SE = 0.85; *p* < 0.0001).

### 3.3. Factors Associated with Health-Related Quality of Life

Table 3 presents the adjusted multivariable linear regression analyses examining the association between anemia group and health-related quality of life (HRQoL), measured by the Physical Component Summary (PCS) and Mental Component Summary (MCS) scores. The regression coefficient (β) represents the adjusted mean difference in the HRQoL score associated with each predictor, relative to its reference category, while holding the other covariates constant (i.e., sociodemographic characteristics, health insurance, health status, physical activity, and chronic conditions).

After adjustment, anemia with depression and/or anxiety was not significantly associated with PCS (β = 1.041, SE = 1.000), but it was independently associated with a 7.09-point lower MCS score compared with anemia alone (β = −7.089, SE = 0.905, *p* < 0.001), indicating a substantial negative association with mental HRQoL.

Younger age was significantly associated with better physical health. Compared with adults aged >64 years, participants aged 22–39 years had PCS scores approximately 7.45 points higher (β = 7.450, SE = 1.621, *p* < 0.001), while those aged 40–49 years had PCS scores approximately 5.65 points higher (β = 5.651, SE = 1.546, *p* < 0.001), whereas age was not significantly associated with MCS. Sex, race/ethnicity, marital status, education, health insurance, prescription drug insurance, and most chronic conditions were not independently associated with either PCS or MCS.

Participants residing in the Midwest had significantly higher PCS scores than those living in the West (β = 2.553, SE = 1.170, *p* < 0.05). Employed participants had PCS scores approximately 3.71 points higher than those who were not employed (β = 3.709, SE = 1.154, *p* < 0.01). Poverty status was associated only with mental health, as participants with near-poor (β = 4.290, SE = 1.392, *p* < 0.01) and middle-income status (β = 4.241, SE = 1.239, *p* < 0.001) had MCS scores that were nearly 4 points higher than that of those living in poverty.

Self-rated general health was one of the strongest predictors of HRQoL. Compared with participants reporting good health, those reporting excellent or very good health had 4-point higher PCS scores (β = 4.008, SE = 1.024, *p* < 0.001), conversely, participants reporting fair/poor health had substantially lower HRQoL scores, 8.86-point lower PCS scores (β = −8.861, SE = 1.056, *p* < 0.001) and 6.87-point lower MCS scores (β = −6.870, SE = 0.936, *p* < 0.001), than those reporting good health. In addition, engaging in physical activity at least three times per week led to 2.61-point higher PCS scores (β = 2.612, SE = 0.881, *p* < 0.01). Among the chronic conditions examined, GERD was associated with a nearly 2-point lower physical health score (β = −2.365, SE = 1.180, *p* < 0.05). No significant associations were observed for heart disease, hypertension, diabetes, hyperlipidemia, asthma, COPD, cancer or arthritis after adjustment.

## 4. Discussion

### 4.1. Prevalence and Comorbidity of Depression and Anxiety

This study examined the association between coexisting depression and/or anxiety and health-related quality of life (HRQoL) among adults with anemia in the United States. Approximately one-third of adults with anemia also had depression and/or anxiety, highlighting the substantial burden of mental health conditions in this population. This finding is consistent with previous studies conducted both globally and in the United States, which have reported a high prevalence of depression and anxiety among individuals with anemia [10,13,16,27]. Individuals with both anemia and depression and/or anxiety reported poorer self-rated health and a higher prevalence of several chronic conditions, including hyperlipidemia, asthma, chronic obstructive pulmonary disease (COPD), arthritis, gastroesophageal reflux disease (GERD), and cancer. The higher prevalence of several chronic conditions and poorer self-rated health among participants with depression and/or anxiety suggests that these individuals may experience a greater overall burden of illness. The coexistence of multiple physical and mental health conditions may increase symptom burden, functional limitations, and healthcare needs, which may in turn contribute to poorer perceived health and HRQoL. This pattern is consistent with previous research showing that these chronic conditions commonly coexist with anemia and contribute to a greater overall disease burden [28,29].

### 4.2. Depression and Anxiety Are Associated with Poorer Mental HRQoL

The most notable finding was that, after adjustment for sociodemographic characteristics, insurance status, general health, physical activity, and chronic conditions, depression and/or anxiety remained independently associated with substantially poorer mental HRQoL but not physical HRQoL. Participants with depression and/or anxiety had MCS scores that were more than seven points lower than that of those with anemia alone, suggesting a clinically meaningful impairment in mental well-being. This association may reflect the direct influence of depression and anxiety on emotional functioning, mood, perceived well-being, and the ability to cope with the demands of living with a chronic condition such as anemia. Symptoms such as fatigue, reduced energy, sleep disturbances, and impaired concentration may also overlap with manifestations of both anemia and depression and anxiety, potentially increasing the overall psychological burden.

This finding underscores the considerable psychological burden experienced by adults living with anemia and highlights the importance of addressing mental health alongside the management of anemia. Our findings are further supported by evidence from a large meta-analysis showing that treatment of anemia with erythropoiesis-stimulating agents significantly improved HRQoL in patients with cancer- and chronic kidney disease-related anemia, with greater improvements in hematocrit associated with greater gains in quality of life [30]. Together, these findings emphasize that optimizing anemia management while addressing coexisting mental health conditions may be essential for improving overall patient well-being.

Our findings are consistent with evidence summarized in a recent review, which reported that treatment of iron deficiency anemia may improve several psychiatric symptoms, including depressed mood, fatigue, anxiety, anhedonia, and sleep disturbances [31]. The review also highlighted that the overlap between symptoms of iron deficiency and depression may contribute to misdiagnosis in some patients [21]. In addition, it suggested that correction of iron deficiency may enhance the effectiveness of antidepressant therapy when iron supplementation is used alongside standard treatment. These observations underscore the importance of considering iron deficiency in the evaluation of patients presenting with depressive symptoms and support an integrated approach to managing anemia and coexisting mental health conditions.

### 4.3. Factors Associated with Physical and Mental HRQoL

The strong association between self-rated health and both PCS and MCS is particularly important. Self-rated health may capture the cumulative effects of symptoms, functional limitations, chronic disease burden, and an individual’s overall perception of their health that may not be fully represented by individual clinical conditions. Moreover, employed participants had higher PCS scores than those who were not employed. This association may reflect better functional capacity, fewer physical limitations, or greater social engagement among individuals who remain employed. Regular physical activity was also associated with higher PCS scores. Although physical activity may contribute to better physical functioning, it may also serve as a marker of better underlying health, and the cross-sectional design prevents determination of the direction of this association.

Participants with depression and/or anxiety had a higher prevalence of several chronic conditions, including hyperlipidemia, asthma, chronic obstructive pulmonary disease (COPD), arthritis, gastroesophageal reflux disease (GERD), and cancer. The accumulation of multiple chronic conditions may increase symptom burden, functional limitations, medication burden, healthcare needs, and the challenges associated with self-management. These factors may contribute to poorer perceived health and lower HRQoL among individuals simultaneously managing anemia and mental health conditions.

The observed pattern may also reflect a bidirectional relationship between mental and physical health. Persistent physical symptoms and limitations associated with chronic diseases may contribute to psychological distress, while depression and anxiety may affect symptom perception, treatment adherence, physical activity, and self-management. However, the present cross-sectional analysis cannot establish the direction of these relationships. Therefore, the observed coexistence of mental health conditions, anemia, and chronic diseases should be interpreted as an indicator of a higher overall burden of illness rather than as evidence of a causal pathway.

Collectively, these findings support a biopsychosocial model of anemia management in which psychological well-being, social determinants of health, and chronic disease burden should be considered alongside hematologic treatment. Addressing anemia alone may not be sufficient to improve patients’ overall quality of life if coexisting mental health conditions remain unidentified and untreated.

### 4.4. Clinical and Policy Implications

The findings have several important clinical and public health implications. First, routine screening for depression and anxiety should be incorporated into the clinical assessment of adults with anemia, particularly among those reporting poor general health or multiple chronic conditions. Early identification of psychological distress may facilitate timely referral for mental health evaluation and treatment.

Second, integrated models of care involving primary care physicians, hematologists, mental health professionals, and pharmacists may improve overall patient outcomes by addressing both the physical and psychological consequences of anemia. Collaborative care approaches have demonstrated benefits in other chronic diseases and may similarly improve quality of life among individuals with anemia.

Third, interventions promoting physical activity, employment support, chronic disease management, and patient self-management may improve physical functioning, while psychological counseling and behavioral interventions may specifically enhance mental HRQoL. Policymakers should also consider improving access to mental health services and prescription drug coverage for vulnerable populations with anemia, particularly those insured through public programs or with limited healthcare resources.

Future longitudinal studies are needed to clarify the temporal relationship between anemia, depression, anxiety, and HRQoL and to determine whether treatment of mental health conditions improves long-term patient-reported outcomes among adults with anemia.

### 4.5. Strengths and Limitations

This study has several strengths. It utilized a large, nationally representative sample of adults with anemia in the United States, allowing the findings to be generalized to the noninstitutionalized adult population. The use of survey weights and complex sampling methods enhances the representativeness of the estimates. In addition, HRQoL was measured using the validated Veterans RAND 12-Item Physical and Mental Component Summary scores, and the analyses accounted for a broad range of sociodemographic, health-related, and clinical covariates, reducing the likelihood of confounding.

Despite these strengths, several limitations should be considered. First, the cross-sectional design precludes establishing causal relationships between depression, anxiety, anemia, and HRQoL. It is not possible to determine whether depression and anxiety contributed to poorer quality of life or whether poorer quality of life increased the likelihood of psychological distress. Second, diagnoses of anemia, depression, anxiety, and chronic conditions were based on self-reported information and may be subject to recall or reporting bias. Third, the dataset did not include information on anemia severity, laboratory parameters (e.g., hemoglobin levels, ferritin concentrations), anemia subtype, treatment status, or duration of illness, all of which may influence quality of life. Fourth, residual confounding from unmeasured variables, including social support, health behaviors, medication adherence, symptom severity, and access to mental healthcare, cannot be excluded. Finally, the study focused on adults with anemia in the United States; therefore, the findings may not be directly generalizable to healthcare systems or populations in other countries.

## 5. Conclusions

Among adults with anemia in the United States, nearly one-third had coexisting depression and/or anxiety. After adjustment, coexisting depression and/or anxiety was independently associated with significantly poorer mental HRQoL but not physical HRQoL. These findings highlight the substantial mental health burden among adults with anemia and underscore the importance of recognizing and addressing coexisting depression and anxiety as part of comprehensive, patient-centered care. Future longitudinal and intervention studies are warranted to determine whether effective management of depression and anxiety, together with optimal anemia treatment, can improve HRQoL. These findings indicate that psychological comorbidity represents an important dimension of the overall burden experienced by adults with anemia. Routine screening for depression and anxiety, timely referral for appropriate psychological care, and integration of mental health services into anemia management may help address unmet patient needs and improve patient-centered outcomes. Future longitudinal and intervention studies are warranted to determine whether effective management of depression and anxiety, together with optimal anemia treatment, can improve HRQoL and reduce the risk of persistent or recurrent psychological distress.

## Figures and Tables

**Table 1:** Characteristics of adults with anemia according to depression/anxiety status (n = 835).

	Total Sample		Anemia Only		Anemia and Depression and/or Anxiety			
	n	Wt.%	n	Wt.%	n	Wt.%	Chi-Square	*p*-Value
All	835	100.0	567	69.3	268	30.7		
Age in Years								
18–39	131	18.7	97	70.5	34	29.5	4.110	0.250
40–49	147	17.8	109	76.7	38	23.3		
50–64	154	17.7	98	68.3	56	31.7		
>64	403	45.8	263	66.3	140	33.7		
Gender								
Women	651	75.5	434	67.4	217	32.6	1.937	0.164
Men	184	24.5	133	74.9	51	25.1		
Race/Ethnicity								
White	440	61.6	271	63.0	169	37.0	24.338	<0.0001
African American	161	16.5	128	83.2	33	16.8		
Latino	179	15.2	125	73.8	54	26.2		
Others	55	6.7	43	81.8	12	18.2		
Marital Status								
Married	354	45.5	261	73.2	93	26.8	3.822	0.148
Wid./Div./Sep.	320	35.3	206	67.0	114	33.0		
Never married	161	19.2	100	63.9	61	36.1		
Education Level								
<High school	75	6.0	52	68.2	23	31.8	4.132	0.248
High school	70	6.0	55	82.6	15	17.4		
>High school	685	87.5	457	68.4	228	31.6		
Region								
Northeast	144	18.4	90	67.3	54	32.7	4.715	0.194
Midwest	181	22.0	120	68.2	61	31.8		
South	325	39.2	228	73.7	97	26.3		
West	185	20.4	129	63.5	56	36.5		
Employment								
Employed	326	44.0	243	73.3	83	26.7	3.173	0.075
Not employed	509	56.0	324	66.1	185	33.9		
Poverty Status								
Poor	186	15.0	114	64.2	72	35.8	1.898	0.594
Near poor	198	21.8	136	68.8	62	31.2		
Middle income	232	30.4	158	69.5	74	30.5		
High income	219	32.8	159	71.7	60	28.3		
Health Insurance								
Private	427	60.1	317	73.4	110	26.6	9.684	0.008
Public	383	37.8	232	62.4	151	37.6		
Uninsured	25	2.1	18	73.2	7	26.8		
Rx Insurance								
Rx insurance	336	46.9	249	74.1	87	25.9	7.257	0.007
No Rx insurance	499	53.1	318	64.9	181	35.1		
General Health								
Excellent/very good	221	32.1	177	77.4	44	22.6	15.207	<0.0001
Good	304	34.9	211	70.8	93	29.2		
Fair/poor	310	33.0	179	59.8	131	40.2		
Physical Activity								
3/week	297	35.6	210	70.7	87	29.3	1.700	0.191
No exercise	537	64.4	356	66.3	81	33.7		
Heart								
Yes	203	24.2	125	63.6	78	36.4	2.889	0.089
No	632	75.8	442	71.1	190	28.9		
Hypertension								
Yes	453	50.2	293	66.1	160	33.9	2.933	0.087
No	382	49.8	274	72.4	108	27.6		
Diabetes								
Yes	216	22.9	142	67.5	74	32.5	0.357	0.550
No	619	77.1	425	69.8	194	30.2		
Hyperlipidemia								
Yes	321	35.5	204	63.8	117	36.2	4.302	0.038
No	514	64.5	363	72.2	151	27.8		
Asthma								
Yes	111	13.2	59	54.1	52	45.9	10.647	0.001
No	724	86.8	508	71.6	216	28.4		
COPD								
Yes	69	7.5	38	53.9	31	46.1	5.008	0.025
No	766	92.5	529	70.5	237	29.5		
Arthritis								
Yes	186	19.2	100	54.0	86	46.0	19.707	<0.0001
No	649	80.8	467	72.9	182	27.1		
GERD								
Yes	193	22.3	114	60.3	79	39.7	7.504	0.006
No	642	77.7	453	71.8	189	28.2		
Cancer								
Yes	93	11.7	53	58.3	40	41.7	4.716	0.030
No	742	88.3	514	70.7	228	29.3		

Categorical variables were summarized using unweighted numbers and weighted raw percentages, and anemia group differences were assessed using chi-square test; “*p*-value represents differences in characteristics between anemia groups”. COPD: chronic obstructive pulmonary disease; GERD: gastroesophageal reflux disease; Wt.: weighted; Rx: medication; Wid./Div./Sep.: widowed, divorced, or separated.

**Table 2:** Weighted means and standard errors of health-related quality of life scores by anemia group among adults with anemia.

	Total Sample			Anemia Only			Anemia and Depression and Anxiety			
	n	Mean	SD	n	Mean	SE	n	Mean	SE	*p*-Value
HRQoL										
PCS	835	40.43	13.14	567	42.78	0.71	268	39.61	0.98	<0.0001
MCS	835	48.78	10.90	567	52.49	0.36	268	44.24	0.85	<0.0001

*p*-value represents significant mean differences by anemia group using survey-adjusted *t*-tests. MCS: Mental Component Summary; PCS: Physical Component Summary; SE: standard error; SD: standard deviation.

**Table 3:** Parameter estimates from adjusted multivariate linear regressions on HRQoL among adults with anemia.

	Health-Related Quality of Life					
	PCS			MCS		
	B	SE	Significance	B	SE	Significance
Anemia Group						
Anemia and depression and anxiety	1.041	1.000		−7.089	0.905	***
Anemia only (Ref.)						
Age in Years						
18–39	7.450	1.621	***	−1.888	1.580	
40–49	5.651	1.546	***	−2.370	1.313	
50–64	1.845	1.296		−0.392	1.264	
>64 (Ref.)						
Gender						
Women	0.075	1.389		0.333	1.038	
Men (Ref.)						
Race/Ethnicity						
African American	0.363	1.166		1.742	1.147	
Latino	1.763	1.244		0.311	1.256	
Others	0.197	1.692		0.375	1.355	
White (Ref.)						
Marital Status						
Married	−0.736	1.029		−0.193	0.995	
Never married	−0.912	1.140		−0.275	1.323	
Wid./DIV./Sep. (Ref.)						
Education Level						
>High school	−1.904	5.358		11.477	9.428	
High school	1.407	5.391		9.792	9.480	
<High school (Ref.)						
Region						
Northeast	−0.194	1.274		−0.333	1.095	
Midwest	2.553	1.170	*	−0.950	1.157	
South	0.938	1.152		−0.929	1.143	
West (Ref.)						
Employment						
Employed	3.709	1.154	**	−0.819	1.128	
Not employed (Ref.)						
Poverty Status						
Near poor	0.753	1.554		4.290	1.392	**
Middle income	−0.853	1.368		4.241	1.239	***
High income	−1.477	1.307		2.340	1.327	
Poor (Ref.)						
Health Insurance						
Private	−0.917	2.046		−0.448	2.218	
Public	−3.092	1.939		−0.692	2.219	
Uninsured (Ref.)						
Rx Insurance						
Rx insurance	−0.864	1.373		−0.202	1.139	
No Rx insurance (Ref.)						
General Health						
Ex/very good	4.008	1.024	***	1.084	0.824	
Fair/poor	−8.861	1.056	***	−6.870	0.936	***
Good (Ref.)						
Physical Activity						
3/week	2.612	0.881	**	0.006	0.756	
No exercise (Ref.)						
Heart						
Yes	−0.254	1.233		1.615	1.024	
Hypertension						
Yes	−1.199	0.995		−0.058	0.932	
Diabetes						
Yes	−1.086	1.228		−1.093	1.048	
Hyperlipidemia						
Yes	0.478	1.073		−0.936	0.953	
Asthma						
Yes	−1.737	1.289		−0.334	1.238	
COPD						
Yes	0.787	1.664		−1.545	1.497	
Arthritis						
Yes	−1.390	1.016		0.188	1.024	
GERD						
Yes	−2.365	1.180	*	0.735	1.085	
Cancer						
Yes	3.092	1.542		0.891	1.130	

Asterisks denote statistical significance in parameter estimates from multivariate linear regressions on health-related quality of life. *** *p* < 0.001; ** 0.001 ≤ *p* < 0.01; * 0.01 ≤ *p* < 0.05. B: regression coefficients; MCS: Mental Component Summary; PCS: Physical Component Summary; Rx: medication; Ref: reference group; SE: standard error; Wid./Div./Sep.: widowed, divorced, or separated; COPD: chronic obstructive pulmonary disease; GERD: gastroesophageal reflux disease.

## Data Availability

The data supporting the findings of this study are publicly available through the Medical Expenditure Panel Survey (MEPS) database at https://meps.ahrq.gov/mepsweb/data_stats/download_data_files.jsp (accessed on 23 May 2026).
